# Administrative embedding and employee health protection in China

**DOI:** 10.3389/fpubh.2025.1719365

**Published:** 2025-12-11

**Authors:** Xiangxu Meng, Yue Wang

**Affiliations:** Business School, Shandong University, Weihai, China

**Keywords:** public health shocks, administrative embedding, employee health protection, private enterprises, COVID-19

## Abstract

**Introduction:**

This study aims to investigate the impact of public health shocks and administrative embedding on employee health protection in privately listed companies in the Shanghai and Shenzhen A-share markets between 2011 and 2021, with a particular focus on the COVID-19 shock.

**Methods:**

This study uses the Difference-in-Differences methodology to examine the impact of administrative embedding on employee health protection in private enterprises following the COVID-19 shock.

**Results:**

Private enterprises with administrative embedding significantly enhanced employee health protection level after experiencing public health shock. The validity of the research conclusions is confirmed through parallel trend test, endogeneity treatment, and robustness checks. This effect is more pronounced in enterprises located in eastern regions, densely populated areas, and larger companies. Increasing attention to employee health protection plays a mediating role, while urban healthcare condition and social security level serve as positive moderating factors.

**Conclusion:**

Private enterprises should recognize the role of administrative embedding in enhancing governance, employee health protection, and social responsibility. Employee involvement is crucial in health policies, with the government encouraging participation through supportive measures. Balancing efficiency and avoiding excessive interference is key to promoting administrative embedding. In future public health crises, this mechanism can strengthen resilience by enabling quick policy support and effective resource mobilization.

## 1 Introduction

Health is a crucial asset, and the COVID-19 shock has raised public awareness of health issues. Private enterprises are no exception, with employee health protection becoming a key concern. This is an essential aspect of corporate social responsibility, and growing emphasis on CSR has heightened the importance of employee health protection.

Private enterprises are a crucial component of China’s economic structure, driving modernization and high-quality development. The “Opinions of the Central Committee of the Communist Party of China and the State Council on Promoting the Development of Private Enterprises” highlights the importance of supporting the non-public sector in fostering economic growth. In the context of China’s corporate governance, administrative embedding refers to the involvement of government departments or party organizations in private enterprises, either through the establishment of party organization or other forms of governance intervention. This influences the enterprise’s decision-making processes, operational strategies, and its fulfillment of social responsibilities. Specifically, administrative embedding does not directly engage in the management of private enterprises, but rather assumes an indirect role by participating in important meetings and decision-making processes within the company. The primary purpose of administrative embedding is to strengthen the linkage between enterprises and the government, thereby encouraging enterprises to more effectively assume social responsibilities and play a constructive role in economic development. In recent years, an increasing number of private enterprises have adopted this governance model, leveraging administrative embedding to enhance their social responsibility and governance capabilities. In response to the public health shock, have these enterprises enhanced employee health protection? What role has administrative embedding played, and what mechanisms have influenced this outcome?

Existing literature has examined factors affecting employee health. Chu (1) found that long working hours negatively impact self-rated health, with more significant deterioration in higher education and male workers. Kivimaki et al. (2) showed that high work stress increases cardiovascular mortality risk, emphasizing long-term health risks. Finkelstein et al. (3) revealed that Medicaid enrollment increased hypertension diagnoses and alleviated depressive symptoms. However, the literature lacks research on the impact of public health shocks on employee health protection. Post-shock, studies have focused on the shock’s economic and social impacts. Guerrieri et al. (4) found that the shock caused output to fall below potential, while Chetty et al. (5) highlighted sharp declines in spending by high-income individuals, leading to layoffs in small businesses. However, no studies have explored the effects of public health shocks on employee health security. There is a growing body of economic literature on the implications of political connections in business (28–33), particularly regarding their value. Political connections help firms secure favorable regulatory conditions (6) and access resources like bank loans (7, 8), increasing firm value (9, 10) and performance (11). In China, administrative embedding plays a key role in enterprise governance and social responsibility. Li et al. (12) find that Party membership positively affects private firms’ performance. Wu et al. (13) show that political connections benefit private enterprises more than state-owned ones, especially in weaker institutional environments. Fan et al. (14) highlight the “double-edged sword” effect of political connections, where they access policy resources but weaken governance, hurting long-term performance. This provides insights into the interaction between state-owned enterprise reform and government-business relations in China. While existing literature explores factors influencing employee health, the socioeconomic impacts of public health shocks, and the effects of political connections on enterprises, there is limited research on the internal governance mechanisms enterprises adopt to address public health shocks. In addition, administrative embedding is also closely related to the literature on social embeddedness. Currently, there is a lack of relevant research on the impact of social embedding on employees’ health protection under public health shocks. Classic studies have pointed out that economic actions are deeply embedded in social relationship networks (15), and embedded relationships enhance organizational action effectiveness through trust, fine-grained information, and joint problem-solving arrangements (16). In China, administrative embedding plays a key role in private enterprises by participating in corporate governance, coordinating resources, supervising policy implementation, and promoting social responsibility. This makes administrative embedding crucial in safeguarding employee health. Therefore, research on how it influences employee health security fills gaps in the literature and offers new insights into corporate strategies in response to public health shocks.

This study examines private listed companies in China’s Shanghai and Shenzhen A-shares from 2011 to 2021, using the difference-in-differences (DID) method to assess the impact of administrative embedding on employee health protection post-COVID-19 shock. The results show that private enterprises with administrative embedding significantly improved employee health protection compared to those without. Robustness checks, parallel trend tests, and endogeneity treatment confirm the reliability of these findings. The impact is stronger in eastern regions, densely populated areas, and large enterprises. Key mechanisms include the focus on safeguarding employee rights and urban medical and social security conditions. Furthermore, administrative embedding has encouraged private enterprises to take on greater social responsibility. This paper contributes by empirically examining the role of administrative embedding in health protection during public health shocks, enhancing the understanding of its effect on private enterprises. It also uses the DID method to identify causal relationships, verifying results through robustness checks. Finally, it provides practical recommendations for improving employee health protection and enhancing corporate governance and government-enterprise relations.

The paper is structured as follows: Section 2 presents the institutional background; Section 3 provides theoretical analysis; Section 4 outlines the research design, including the econometric model, variable construction, data sources, and descriptive statistics; Section 5 presents the empirical analysis; Section 6 discusses mechanism tests and further analysis; Section 7 concludes with policy recommendations.

## 2 Theoretical background

### 2.1 Administrative embedding in private enterprises

Administrative embedding represents a distinctive characteristic of corporate governance in China. In state-owned enterprises, it is an indispensable and essential component. However, in private enterprises, administrative embedding is characterized by a degree of voluntariness. According to surveys, from 2002 to 2018, the coverage rate of administrative supervision embedded in private enterprises in China is continuously increasing, from 27.42% in 2002 to 48.31% in 2018. The proportion of private enterprises with administrative embedding has now reached nearly half, significantly influencing business operations, corporate governance, and the fulfillment of social responsibilities. Within enterprises, administrative supervision embedding can, on the one hand, foster unity and strengthen employee cohesion, and on the other hand, play a critical role in guiding, supporting, and supervising the lawful and efficient operation of enterprises, optimizing corporate governance structures, and ensuring the fulfillment of social responsibilities. Administrative embedding within enterprises acts as a crucial guide and supervisor in the development of the enterprise. From the perspective of social embeddedness theory, administrative embedding is not just about government oversight of private enterprises but is also a process that promotes corporate behavior through social networks. According to Granovetter (15), economic actions are not entirely driven by market forces, but are deeply embedded in social structures and relationships. Granovetter argues that economic behavior is “embedded” in ongoing interpersonal interactions, rather than being the result of isolated individual decisions. In this framework, administrative embedding influences not only corporate compliance and governance efficiency but also fosters social responsibility, especially during crises such as the COVID-19 shock. Additionally, Uzzi (16) further suggests that interfirm social embeddedness enhances economic actions through trust, fine-grained information, and joint problem-solving arrangements. Through close relationships with the government and other enterprises, private companies can gain better resources, information, and support, allowing for more effective coordination and cooperation in a complex economic environment. Therefore, administrative embedding brings a dual benefit: it ensures that enterprises comply with laws and policies while strengthening social network relationships, improving their market adaptability and competitiveness.

In conclusion, administrative embedding, through its embedded social networks, not only enhances governance efficiency but also plays an essential role in helping private enterprises respond to crises, fulfill social responsibilities, and promote sustainable development.

### 2.2 Employee health protection

As a component of employee welfare, employee protection is attracting more and more attention (17). Employee protection is a key component of corporate social responsibility. Ensuring the well-being of employees not only contributes to the sustainable development of enterprises but also plays a crucial role in maintaining social stability and promoting economic prosperity. First, protecting employees from unemployment or unfair treatment helps maintain social stability, prevent social unrest, and promote societal harmony. Employees are valuable assets to any organization, and their health, rights, and quality of life directly affect the productivity of the enterprise and the overall stability of society (18).

Within the framework of employee protection, employee health protection is particularly prominent. Since the COVID-19 shock, both government and enterprises have significantly increased their focus on employee health protection. Employee health protection involves safeguarding employees’ physical and mental health, including ensuring their safety, health, and welfare in the workplace. Employee health protection is not only about caring for employees’ personal health but also serves as an important manifestation of a company’s commitment to fulfilling its social responsibility. With the increasing demands for employee health protection in the post-shock period, employee health protection has become an integral part of both corporate competitiveness and corporate social responsibility.

## 3 Hypothesis

According to Granovetter’s (15) social embeddedness theory, economic behavior does not occur in isolation, but is deeply embedded within social networks of relationships. Granovetter (15) posits that the effectiveness and efficiency of economic actions often depend on the interactions between individuals or enterprises within these social networks. During the COVID-19, administrative embedding strengthened social connections and supervisory networks between the government and enterprises, enhancing their capacity to coordinate and respond to public health crises. Administrative embedding is not merely a tool for government supervision; it also promotes more effective employee health protection by reinforcing both internal and external links within enterprises, thereby improving information sharing and trust mechanisms. Especially during the COVID-19 shock, businesses were able to swiftly implement health protection measures, mitigate risks, and ensure the safety and health of their employees through existing social networks and government support. This theoretical framework helps us understand that the COVID-19 shock is not only a health crisis but also exposes vulnerabilities within corporate governance systems and public health systems. Social embedding provides theoretical support for understanding this corporate response pattern, explaining how enhancing the connections between enterprises and social networks can result in more effective corporate governance and employee protection during public health crises.

In combating the COVID-19, the Communist Party of China played a pivotal role, demonstrating exemplary leadership. In late 2019, a novel coronavirus was detected in Wuhan, initially attracting little attention. However, as the virus spread rapidly, the COVID-19 escalated, leading to widespread national concern. In January 2020, Wuhan implemented a citywide lockdown, marking China’s first such response. Other cities quickly followed with measures to control movement, trace the virus, and deploy expert teams. Once person-to-person transmission was confirmed, strict nationwide measures were implemented. The devastating impact of the COVID-19 left a lasting impression, significantly raising public health awareness and prompting a heightened focus on strengthening health protection systems and improving public health infrastructure.

Similarly, private enterprises also need guidance, support, and oversight through administrative embedding. In private enterprises, unionization levels are generally low, and the role of unions is relatively weak, resulting in insufficient institutional support for employees in areas such as labor contract signing, welfare protection, and health protection. Administrative embedding helps address this deficiency by enhancing the protection of employees’ legal rights and advancing corporate health management. The existing research indicates that administrative embedding can significantly improve employee labor contract signing rates, training investment, and the welfare levels of workers (19). Specifically, administrative embedding can coordinate labor relations and promote collective wage bargaining through leadership of unions and workers’ representative assemblies, thereby improving employee welfare and health protection. The presence of administrative embedding fosters more equitable labor distribution plans and the implementation of health and safety systems, as it ensures the involvement of party organizations or government representatives in management meetings. Additionally, administrative embedding, through promoting the party’s long-term development goals and policy ideas, can curb inequitable distribution behaviors within enterprises and safeguard employees’ health rights. As a crucial component of corporate governance and a core dimension of corporate social responsibility, employee health protection is of critical importance to private enterprises and represents a key area where administrative embedding plays a critical role. Therefore, administrative embedding enhances employees’ recognition and participation in health protection measures, thereby encouraging enterprises to more actively upgrade their employee health protection practices following the COVID-19 shock.

Based on the above theoretical analysis, this paper proposes hypothesis 1:

*Hypothesis* 1: Following the shock of the COVID-19, private enterprises with administrative embedding are more likely to enhance employee health protection levels.

The theory of attention allocation originated in psychology, where it describes the selective focus individuals adopt when processing multiple stimuli (20). Biological research further demonstrates that organisms consciously prioritize certain inputs in complex environments, reflecting the limited capacity and structured allocation of attentional resources (21). Simon (22) was the first to introduce the concept of attention into the fields of management and organizational behavior, laying the foundation for discussions on bounded rationality. He argued that decision-makers, constrained by limited cognitive capacity, cannot process all available information and must therefore focus selectively. Attention, as a scarce resource, is continuously allocated and redirected during decision-making. Subsequently, Kahneman (23), from the perspective of cognitive psychology, proposed the “attention resource theory,” emphasizing that information recognition, processing, and judgment rely on limited cognitive resources. Individuals must dynamically allocate these resources among different tasks, and the efficiency of this allocation directly determines the quality of their judgments and behavioral performance. In organizational studies, Ocasio (24) advanced the Attention-Based View, which posits that managerial attention is shaped not only by cognitive constraints but also by organizational structures and external contexts. This framework has been widely used to explain how firms prioritize issues and allocate resources under complexity. Administrative embedding has not only enhanced private enterprises’ attention to employee health protection but also directed and strengthened this focus, making employee health a priority in corporate decision-making. Administrative embedding effectively channels attention, elevating employee health issues to the strategic level of corporate governance. This focus prompts enterprises to place greater emphasis on employee health, prioritize the implementation of health protection measures, and, consequently, improve both employee welfare and the company’s overall social responsibility. Therefore, by intensifying the focus on employee health, administrative embedding has significantly improved health protection measures in private enterprises and encouraged a proactive response from businesses to employee protection during public health crises.

Organizations do not exist in isolation; their survival and development heavily depend on the critical resources provided by the external environment. According to Resource Dependence Theory (25), organizational behavior is significantly influenced by the ability to acquire key external resources. To secure these resources, organizations adjust their strategies and structures to adapt to external environmental conditions. Urban healthcare infrastructure and social security systems are key external resource factors that influence a company’s ability to protect employee health. In regions with well-developed urban healthcare services and robust social security systems, enterprises can more easily access abundant public health resources and leverage external professional expertise to enhance employee health protection. Additionally, a comprehensive social security environment strengthens the institutional linkage between the government and enterprises, improving the effectiveness of administrative embedding mechanisms, and encouraging companies to prioritize employee health protection as a core governance issue. In contrast, in areas with scarce medical resources or weak social security systems, enterprises may face difficulties in effectively advancing health protection measures, even if they have administrative embedding mechanisms in place, due to resource limitations.

Based on the above analysis, this paper proposes hypothesis 2:

*Hypothesis* 2: Increasing attention to employee health protection is a crucial mediating variable through which administrative embedding enhances the level of employee health protection in private enterprises. Urban healthcare conditions and social security levels play a positive moderating role in the process of administrative embedding promoting the improvement of employee health protection levels in enterprises.

Population mobility is a key determinant in the spread of the COVID-19. Areas characterized by higher population density and more frequent population movement are often disproportionately affected by COVID-19, leading to heightened public concern for health protection. In China, the eastern regions typically experience higher population inflows, greater population densities, and are more acutely affected by the COVID-19. Consequently, individuals in these regions are likely to place greater emphasis on health protection matters. Furthermore, the eastern regions and densely populated areas are generally more economically developed and possess more robust systems, which may foster stronger and more standardized efforts in employee health protection. Following the COVID-19, private enterprises with administrative embedding may exhibit greater willingness and capacity to enhance employee health protection levels.

At the same time, Economies of Scale Theory posits that large enterprises typically possess more resources, funding, and technical support, enabling them to implement more effective measures in response to public health crises. According to Panzar and Willig (26), as a business expands, its unit costs decline, which allows the enterprise to allocate resources more efficiently and respond more swiftly to external challenges. Due to their larger scale, enterprises have greater financial and material resources, enabling them to adjust and implement health protection strategies more quickly, thus enhancing their capacity to respond to public health emergencies. These companies can leverage their substantial financial resources to procure medical equipment, establish employee health protection programs, provide psychological support services, and improve workplace safety measures. Moreover, Chandler (27) emphasizes that large enterprises typically possess more advanced management systems and larger specialized teams, which enable them to manage health protection resources and measures more effectively, thereby strengthening their overall crisis response capacity.

Based on the preceding theoretical analysis, this paper puts forward hypothesis 3:

*Hypothesis* 3: In regions with higher population densities and more profound impacts from the COVID-19 shock, as well as in larger enterprises, administrative embedding within private enterprises is more likely to foster the enhancement of employee health protection levels.

## 4 Research design

### 4.1 Modeling

This paper empirically tests the above research hypothesis using a sample of privately listed companies on the Shanghai and Shenzhen A-shares over the period from 2011 to 2021. The econometric regression model is outlined as Equation 1:


$$y_{\mathrm{it}}=\beta_{0}+\beta_{1}\text{covid}19_{t}\times \mathrm{AS}E_{i}+\beta_{2}X_{\mathrm{it}}+\mu_{i}+\mu_{t}+\varepsilon_{\mathrm{it}}$$ (1)

In the econometric model specified, y_it_ represents the level of employee health protection for private enterprise i at time t; covid19_t_ is a dummy variable indicating whether the time period occurs after the COVID-19 shock, coded as 1 if the time period occurs after the shock, and 0 otherwise; ASE_i_ is a dummy variable representing whether private enterprise i has administrative supervision embedding, defined by the establishment of an administrative supervision embedded organization. Within the enterprise. If enterprise i has an administrative supervision embedded organization in place, it is coded as 1, otherwise 0; X_it_ includes time-varying variables at the enterprise level that may influence the health protection levels of employees in private enterprises, used as control variables. These include enterprise size, debt-to-asset ratio, operating income, and net profit; μ_i_ represents the individual fixed effects of the enterprise, accounting for factors that do not vary over time and influence employee health protection at the enterprise level; μ_t_ represents the time fixed effects, accounting for macroeconomic factors that do not change with the enterprise’s characteristics and influence employee health protection; ε_it_ is the random error term, and clustering at the enterprise level is used for regression.

### 4.2 Data

The research sample used in this study consists of enterprise-level panel data from privately held A-share listed companies on the Shanghai and Shenzhen stock exchanges in China, covering the period from 2011 to 2021. The data was first processed by excluding companies with ST, *ST, and SST labels during the sample period; financial companies; companies that had been delisted; companies listed within the same year; companies issuing both A-shares and B-shares; and companies with abnormal data or significant data missing. After processing the data, a total of 34,260 research samples were retained.

### 4.3 Variables

Employee health protection: The employee health protection variable is binary, based on corporate annual reports. It is coded as 1 if the company discloses investments in employee health protection (e.g., health check-ups, health-related accident liabilities, safety measures) and 0 otherwise. Disclosure of health check-ups, occupational disease funds, safety training, or no safety accidents results in a value of 1; otherwise, it is coded as 0.

COVID-19: In this study, the COVID-19 dummy variable indicates whether the company is in a post-shock period. It takes the value of 1 if the company is in the period following the outbreak of the COVID-19, and 0 otherwise. Since the COVID-19 in China began in late 2019 and subsequently spread nationwide, this study assigns the value of 1 to the COVID-19 dummy variable for periods after 2019, and 0 otherwise.

Administrative embedding: If private enterprise i has established an administrative supervision embedded organization, the variable is coded as 1; otherwise, it is coded as 0. This data is collected by crawling the contents of the annual reports of listed companies on the Shanghai and Shenzhen A-share markets and the minutes of administrative organization meetings, using keywords such as ‘Party Committee,’ ‘General Party Branch Committee,’ ‘Party Branch,’ and ‘Party Branch Committee,’ to identify whether the enterprise had a party organization Then, manual verification is conducted and the above data is supplemented to ensure the completeness of the data. Additionally, a robustness check is conducted by using the age of the administrative supervision embedded organization’s establishment in private enterprises.

Control variables: This study controls individual fixed effects at both the enterprise and time levels, accounting for time-invariant factors affecting employee health protection. It also controls for time-varying enterprise-level factors, such as size, debt-to-asset ratio, operating income, and net profit. Larger enterprises, with more resources, are more likely to enhance employee health protection to attract talent and gain positive social evaluation. Enterprise size is measured by the natural logarithm of total assets. A higher debt-to-asset ratio suggests poorer financial conditions, reducing the ability to improve health protection. Operating income and net profit indicate business performance and profitability, with higher values increasing the enterprise’s ability to enhance employee health protection. The data are sourced from the CSMAR database.

### 4.4 Descriptive statistics

The descriptive statistics in Table 1 show that the average employee health protection level in private enterprises is 0.229, with a standard deviation of 0.420, indicating significant variation and suggesting an underdeveloped health protection system in China’s private sector. The COVID-19 shock variable has a mean of 0.459, indicating that 46% of the sample was affected by the COVID-19. About 36% of private enterprises have implemented administrative embedding, as indicated by the mean of 0.359 for the administrative supervision embedded organization establishment variable. Additionally, the average enterprise size is 22.366, the mean debt-to-asset ratio is 0.411, and the average operating income is 21.718. These descriptive statistics align with real-world conditions and existing literature.

**Table 1:** Descriptive statistics.

Variables	N	Mean	SD	Min	Median	Max
Employee health protection	34,260	0.229	0.420	0	0	1
COVID-19	34,260	0.459	0.498	0	0	1
Administrative embedding	34,260	0.359	0.480	0	0	1
Enterprise size	34,260	22.366	1.380	15.979	22.205	26.684
Debt-to-asset ratio	34,260	0.411	1.011	0.007	0.401	178.346
Operating income	34,111	21.718	1.567	9.044	21.586	26.556
Net profit	31,484	19.340	1.665	10.338	19.240	24.137

## 5 Empirical results and analysis

### 5.1 Baseline regression

The baseline regression results in Table 2 show that, in all columns (1) to (5), the coefficient of the interaction term between the COVID-19 impact and administrative embedding is significant at the 1% level. This suggests that, after the shock, private enterprises with administrative embedding significantly improved employee health insurance compared to those without it. In column (5), which includes all control variables, the results indicate that these enterprises improved employee health insurance by 9.5%. Additionally, larger enterprise size and stronger profitability are associated with better employee health insurance coverage.

**Table 2:** The baseline regression.

Variables	Employee health protection				
	(1)	(2)	(3)	(4)	(5)
Covid19_t_*ASE_i_	0.089^***^ (0.030)	0.097^***^ (0.029)	0.097^***^ (0.029)	0.097^***^ (0.029)	0.095^***^ (0.031)
Enterprise size		0.064^***^ (0.014)	0.064^***^ (0.014)	0.052^**^ (0.021)	0.048^*^ (0.026)
Debt-to-asset ratio			0.000 (0.001)	0.000 (0.001)	−0.025 (0.024)
Operating income				0.015 (0.014)	−0.002 (0.020)
Net profit					0.026^**^ (0.011)
Constant	0.216^***^ (0.005)	−1.226^***^ (0.321)	−1.227^***^ (0.323)	−1.269^***^ (0.322)	−1.307^***^ (0.373)
Firm FE	Yes	Yes	Yes	Yes	Yes
Year FE	Yes	Yes	Yes	Yes	Yes
Observations	34,171	34,171	34,171	34,022	31,235
Adj R-square	0.710	0.713	0.713	0.713	0.722

The values in parentheses represent robust standard errors clustered at the firm level. *, *, and *** denote significance at the 10, 5, and 1% levels, respectively.

### 5.2 Parallel trend test

To draw reliable conclusions using the difference-in-differences (DID) method, the parallel trend assumption must be satisfied. This assumption requires that, before the external shock, the treatment and control groups follow identical trends in the variable of interest. If the treatment and control groups exhibit identical trends prior to the shock, but diverge significantly afterward, it indicates a causal relationship between the shock and the variable. In this study, the COVID-19 shock serves as policy shock. The treatment group includes private enterprises with administrative embedding, while the control group consists of those without. To test the parallel trends, we use the event study approach, which statistically examines differences between the two groups over time, considered more reliable than the graphical approach. The regression model for this test is as Equation 2:


$$y_{\mathrm{it}}=\beta_{0}+\mathrm{AS}E_{i}\times \sum_{\tau =-3,\tau \neq -1}^{2}\mathrm{yea}r_{\tau }+\beta_{1}X_{\mathrm{it}}+\mu_{i}+\mu_{t}+\varepsilon_{\mathrm{it}}$$ (2)

In this model, y_it_ retains the same definition as in the baseline regression equation, denoting the health insurance level of employees in enterprise i at time t. ASE_i_ represents whether private enterprise iii has implemented administrative embedding; if enterprise i has established an administrative supervision embedded organization, it is assigned the value 1; otherwise, it takes the value 0. Year_τ_ is a time dummy variable; given that the COVID-19 shock emerged at the end of 2019, we treat 2018 as the reference year, and assign periods prior to 2016 as period −3. X_it_ represents factors that may affect the health insurance level of employees in private enterprise i at time t, which are included as control variables, consistent with those in the baseline regression. μ_i_ represents the firm-specific fixed effects, μ_t_ denotes the time fixed effects, and ε_it_ denotes the robust error term clustered at the firm level.

**Figure 1:**
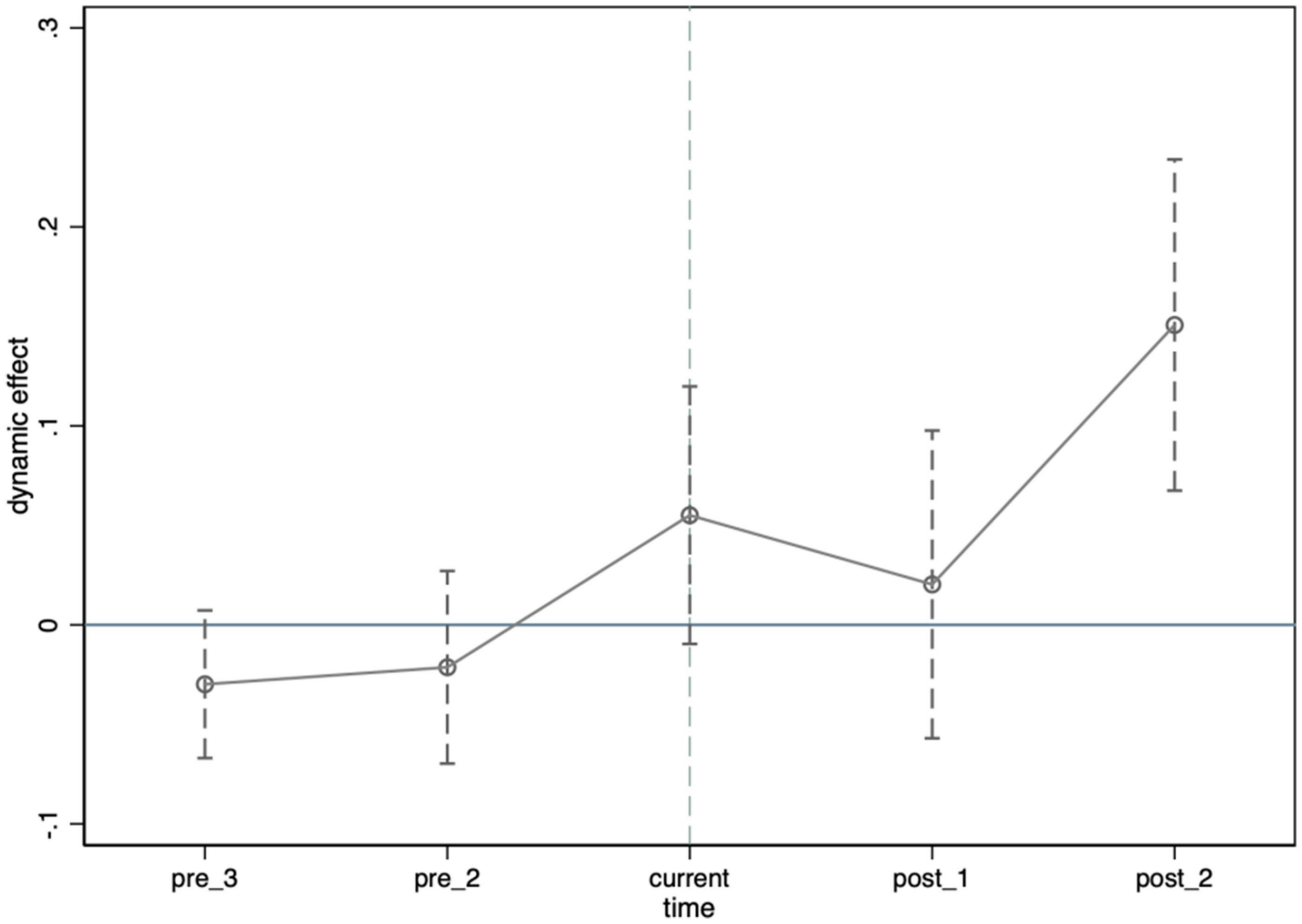
Parallel trends test plot.

The trend graph for the parallel trend test (Figure 1) and the regression results (Table 3) show that, before the shock, there was no significant difference in employee health insurance levels between the treatment (private enterprises with administrative supervision embedded) and control (without embedding) groups. After the COVID-19 shock, enterprises with administrative embedding showed significantly improved employee health protection, indicating that the shock prompted these enterprises to focus more on safeguarding employee health. The results confirm that the parallel trends assumption holds, and the baseline regression results remain robust.

**Table 3:** Parallel trend test.

Variables	Employee health protection
Pre_3	−0.030 (0.019)
Pre_2	−0.021 (0.025)
Current	0.055^*^ (0.033)
Post_1	0.020 (0.039)
Post_2	0.151^***^ (0.042)
Control variables	Yes
Firm FE	Yes
Year FE	Yes
observations	31,235
Adj R-square	0.724

The values in parentheses represent robust standard errors clustered at the firm level. *, *, and *** denote significance at the 10, 5, and 1% levels, respectively.

### 5.3 Endogeneity treatment

Given that enterprises with higher employee health insurance levels may be more inclined to engage in administrative embedding, this study addresses potential endogeneity by using an instrumental variable (IV) and applying the two-stage least squares (2SLS) method. Specifically, the IV is the number of senior executives with more than three administrative supervision embedded organization members. According to the Party Constitution, any enterprise with more than three party members among senior executives must establish an administrative supervision embedded organization. This factor is strongly linked to the establishment of such an organization, but does not directly affect employee health insurance levels, meeting the relevance and exclusion conditions for a valid IV.

The 2SLS results, shown in Table 4, indicate a significant first-stage correlation between the IV and the endogenous variable, satisfying the relevance condition. The second-stage regression results align with the baseline findings, confirming that after the COVID-19 shock, administrative embedding significantly improved employee health insurance levels. These results remain robust after addressing endogeneity concerns.

**Table 4:** Two-stage least squares regression.

Variables	Covid19_t_*ASE_i_	Employee health protection
Covid19_t_*ASE_i_		0.234^**^ (0.109)
Instrumental variable	0.664^***^ (0.022)	
Control variables	Yes	Yes
Firm FE	Yes	Yes
Year FE	Yes	Yes
Observations	31,235	31,235
Adj R-square		0.00707
F statistic	950.74	
LM statistic	26.772 (0.000)	

The values in parentheses represent robust standard errors clustered at the firm level. *, *, and *** denote significance at the 10, 5, and 1% levels, respectively.

### 5.4 Robustness check

To further assess the robustness of the baseline regression results, we conducted several robustness checks, including shortening the time window of the sample, using only the sample of companies listed on the main board, modifying the measurement methods of the explanatory and dependent variables, applying the PSM-DID method, and controlling for policy interference at the city and industry levels. Furthermore, we conducted a placebo test by randomly assigning the exogenous policy shock variable to the sample.

#### 5.4.1 Change the sample

First, we shortened the sample period prior to the COVID-19 shock and conducted econometric regression tests using the sample from 2017 to 2021. The results of this test are shown in column (1) of Table 5. Next, due to the more standardized and complete information disclosure of companies listed on the main board, we further selected a sample of private enterprises listed on the main board for robustness checks. The results of this test are shown in column (2) of Table 5.

**Table 5:** Robustness test results for changing the sample.

Variables	Shorten the time window	Main board
	(1)	(2)
Covid19_t_*ASE_i_	0.081^***^ (0.029)	5.267^***^ (1.684)
Control variables	Yes	Yes
Firm FE	Yes	Yes
Year FE	Yes	Yes
Observations	21,780	22,823
Adj R-square	0.766	0.722

The values in parentheses represent robust standard errors clustered at the firm level. *, *, and *** denote significance at the 10, 5, and 1% levels, respectively.

The results in column (1) of Table 5 demonstrate that, even after shortening the sample period, private enterprises that engaged in administrative embedding continued to significantly enhance the health insurance levels of their employees following the COVID-19 shock. The results in column (2), based on the sample of private listed companies on the main board, are consistent with those in column (1) and further suggest that, after the shock, private enterprises that engaged in administrative embedding placed greater emphasis on improving the health insurance levels of their employees. The robustness checks in both column (1) and column (2) validate the robustness of the baseline regression results.

#### 5.4.2 Replacement of key variables

Subsequently, we modified the measurement methods for the key explanatory and dependent variables. Specifically, we employed standardized employee health insurance coverage as the dependent variable (with data sourced from CSMAR) and used the age of the administrative supervision embedded organization within private enterprises as a measure for the administrative embedding variable, testing the robustness of the baseline regression results. The results of these tests are presented in columns (1) and (2) of Table 6.

**Table 6:** Robustness test results for replacing key variables.

Variables	Employee health protection (standardized)	Employee health protection
	(1)	(2)
Covid19_t_*ASE_i_	4.739^***^ (1.541)	
Covid19_t_*ASEyear_i_		0.225^*^ (0.123)
Control variables	Yes	Yes
Firm FE	Yes	Yes
Year FE	Yes	Yes
Observations	31,235	31,235
Adj R-square	0.722	0.720

The values in parentheses represent robust standard errors clustered at the firm level. *, *, and *** denote significance at the 10, 5, and 1% levels, respectively.

The regression results in columns (1) and (2) of Table 6 indicate that, following the shock, Private enterprises with administrative embedding significantly enhanced the health insurance coverage of their employees. Moreover, the older the administrative supervision embedded organization within private enterprises, the greater the impact. The robustness checks, using the substituted measurement methods for key variables, confirm the robustness of the baseline regression results.

#### 5.4.3 PSM-did

To enhance the comparability between the experimental and control group samples, we next perform a robustness check on the baseline regression results using the Propensity Score Matching-Difference in Differences (PSM-DID) method. The propensity score matching method ensures greater similarity and comparability between the experimental and control groups, thereby providing a more precise causal relationship between the exogenous shock and the outcome variable. Specifically, this study applies the radius matching method for propensity score matching, utilizing matching radii of 0.01, 0.001, and 0.0001. The control variables from the baseline regression are selected as matching variables, and the logit method is employed to estimate the propensity scores. The results confirm that the matching procedure meets the balance requirement. The results from the econometric regression utilizing the PSM-DID method are presented in Table 7. The regression results in Table 7 indicate that the findings from the PSM-DID method are consistent with the baseline regression results, both suggesting that after the shock, private enterprises that established administrative supervision embedded organizations significantly improved the health insurance coverage for their employees. The robustness check using the alternative econometric method further confirms the robustness of the baseline regression results.

**Table 7:** Robustness test results for PSM-DID.

Variables	Radius matching		
	(0.01)	(0.001)	(0.0001)
Covid19_t_*ASE_i_	4.438^***^ (1.525)	4.205^***^ (1.539)	3.005^*^ (1.534)
Control variables	Yes	Yes	Yes
Firm FE	Yes	Yes	Yes
Year FE	Yes	Yes	Yes
Observations	31,143	29,723	19,767
Adj R-square	0.726	0.725	0.780

The values in parentheses represent robust standard errors clustered at the firm level. *, *, and *** denote significance at the 10, 5, and 1% levels, respectively.

#### 5.4.4 Excluding other policy interferences

To account for potential city- or industry-level policy factors affecting employee health insurance coverage, we control for city and industry fixed effects in the regression. The results are shown in Table 8. Column (1) includes city fixed effects, column (2) includes industry fixed effects, and column (3) includes both. The results in column (1) show that, after adding city fixed effects, the coefficient for the interaction term between the COVID-19 shock and administrative embedding remains significantly positive, indicating that private enterprises engaged in administrative embedding improved employee health insurance coverage post-shock. This coefficient size matches the baseline regression, confirming robustness. Column (2) shows that even after accounting for industry-level policy influences, the coefficient remains significantly positive, supporting the baseline results. Column (3) confirms that including both city and industry fixed effects still shows a significantly positive coefficient, reinforcing the robustness of the baseline results. Overall, controlling city and industry factors consistently supports the baseline results.

**Table 8:** Robustness check results excluding interference from other policy factors.

Variables	Employee health protection		
	(1)	(2)	(3)
Covid19_t_*ASE_i_	0.118^***^ (0.031)	0.099^***^ (0.030)	0.130^***^ (0.029)
Control variables	Yes	Yes	Yes
Firm FE	Yes	Yes	Yes
Year FE	Yes	Yes	Yes
City FE	Yes		Yes
Industry FE		Yes	Yes
observations	30,177	31,156	30,090
Adj R-square	0.824	0.793	0.855

The values in parentheses represent robust standard errors clustered at the firm level. *, *, and *** denote significance at the 10, 5, and 1% levels, respectively.

#### 5.4.5 Placebo test

**Figure 2:**
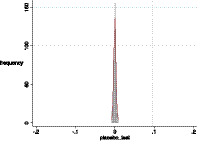
Placebo test.

Furthermore, we randomly assigned the exogenous shock variable within the study sample and performed an econometric regression analysis using health insurance coverage for employees in private enterprises as the dependent variable, with the virtual exogenous shock variable as the independent variable. This process was repeated 5,000 times to obtain a distribution of the regression coefficients for the virtual exogenous shock variable, which was then compared with the actual regression coefficient of 0.095 for the exogenous shock variable to conduct the placebo test. The results of the econometric regression are presented in Figure 2.

The placebo test results presented in Figure 2 reveal that the coefficients for the virtual exogenous shock variable are close to zero. A significant difference exists between the coefficient obtained from regressing health insurance coverage for employees in private enterprises on the virtual exogenous shock variable and the actual regression coefficient of the exogenous shock variable. This suggests that unobserved variables and non-random factors do not distort the estimation results, thereby confirming the robustness of the baseline regression outcomes.

### 5.5 Heterogeneity

The impact of the COVID-19 shock differs across regions and types of private enterprises, with notable variations in their motivations and willingness to establish administrative supervision embedded organizations. Furthermore, there are substantial differences in the institutional environment and economic conditions across regions. Consequently, the effects of the COVID-19 shock and the establishment of administrative supervision embedded organizations in private enterprises on employee health insurance coverage may differ substantially across regions and types of enterprises. To examine this, we further conduct a heterogeneity test to assess the impact of the COVID-19 shock and the establishment of administrative supervision embedded organizations in private enterprises on employee health insurance coverage.

#### 5.5.1 Regional heterogeneity

Firstly, the impact of the COVID-19 shock varies across regions, with substantial differences in the emphasis placed on administrative embedding by private enterprises in each region. Furthermore, there are considerable differences in economic development levels and the robustness of institutional frameworks across regions. Moreover, in private enterprises across regions, the level of employee rights protection and the attention given to employee health vary significantly. Therefore, this study initially categorizes the research sample into private enterprises from the eastern, central, western, and northeastern regions, and performs separate econometric regression analyses for each category. This methodology examines the regional heterogeneity in the impact of the COVID-19 and administrative embedding on health insurance coverage for employees in private enterprises. The results of the analysis are presented in Table 9.

**Table 9:** Heterogeneity results by region.

Variables	Eastern	Central	Western	Northeastern
	(1)	(2)	(3)	(4)
Covid19_t_*ASE_i_	6.042^***^ (1.752)	2.524 (3.749)	2.757 (4.888)	−0.847 (5.497)
Control variables	Yes	Yes	Yes	Yes
Firm FE	Yes	Yes	Yes	Yes
Year FE	Yes	Yes	Yes	Yes
observations	23,484	3,609	3,305	821
Adj R-square	0.733	0.750	0.681	0.587

The values in parentheses represent robust standard errors clustered at the firm level. *, *, and *** denote significance at the 10, 5, and 1% levels, respectively.

Based on the results presented in Table 9, it is evident that in private enterprises located in the eastern region, following the impact of the COVID-19 shock, employee health insurance coverage in enterprises with administrative embedding significantly improved compared to those without administrative embedding. In contrast, no significant difference in employee health insurance coverage was observed between enterprises with and without administrative embedding in the central, western, and northeastern regions after the shock. The higher economic development levels and more robust institutional environment in the eastern region likely result in greater emphasis on administrative embedding and employee health insurance coverage. Consequently, the positive effect of administrative embedding on employee health insurance coverage was fully realized in eastern region private enterprises following the shock. In contrast, in the central, western, and northeastern regions, with their relatively lower levels of economic development and weaker institutional frameworks, the potential benefits of the COVID-19 shock and administrative embedding on employee health insurance coverage were not fully realized. Therefore, it can be concluded that the external economic and institutional environment plays a critical role in the effectiveness of administrative embedding in private enterprises.

#### 5.5.2 Population density heterogeneity

Furthermore, in regions with higher population density, the spread of COVID-19 is more pronounced, and the challenges associated with prevention and control are greater, resulting in more significant impacts on the local economy and society. In contrast, in regions with lower population density, the spread and control of the shock are comparatively easier, and the impact on the local economy and society is less severe. Therefore, we further investigate the differences in the role of the COVID-19 shock and administrative embedding in private enterprises in enhancing employee health insurance coverage across cities with varying population densities. Given that provincial capital cities in China are typically population hubs with higher population densities, we classify provincial capital cities as high population density areas and non-provincial capital cities as low population density areas. The results of the heterogeneity test for cities with high and low population densities are presented in Table 10. Column (1) presents the econometric regression results for cities with high population density, specifically using the sample of provincial capital cities, while Column (2) presents the results for cities with low population density, specifically using the sample of non-provincial capital cities.

**Table 10:** Heterogeneity results by population density.

Variables	Provincial capital city	Non-provincial capital city
	(1)	(2)
Covid19_t_*ASE_i_	8.071^***^ (2.754)	2.394 (1.816)
Control variables	Yes	Yes
Firm FE	Yes	Yes
Year FE	Yes	Yes
observations	12,502	18,720
Adj R-square	0.710	0.736

The values in parentheses represent robust standard errors clustered at the firm level. *, *, and *** denote significance at the 10, 5, and 1% levels, respectively.

Based on the results in Column (1), it can be observed that in high population density cities (i.e., provincial capital cities), following the COVID-19 shock, private enterprises with administrative embedding significantly enhanced the health insurance coverage for their employees. In contrast, in cities with lower population density (i.e., non-provincial capital cities), private enterprises with administrative embedding did not significantly enhance the health insurance coverage for their employees compared to those without administrative embedding. On one hand, this may be attributed to the greater effect of the COVID-19 shock in high population density areas, prompting governments, enterprises, and individuals in these areas to place greater emphasis on health insurance coverage. On the other hand, high population density areas are typically more economically developed, with a more well-established institutional environment, prompting private enterprises to place greater importance on administrative embedding and the enhancement of employee health insurance coverage. Therefore, it is evident that strengthening administrative embedding in high population density areas will result in greater benefits. Moreover, it is evident that in economically developed regions with more comprehensive institutional environments, the positive impact of administrative embedding in private enterprises is more effectively realized.

#### 5.5.3 Heterogeneity of firm size

Considering that private enterprises of different sizes exhibit significant differences in their capacity to respond to the impact of the COVID-19 shock, implement administrative embedding, and enhance employee health insurance coverage, it is crucial to empirically examine the variations in the effects of the COVID-19 shock and administrative embedding on employee health insurance coverage across enterprises of different sizes. To this end, this study classifies the research sample into large and small enterprises based on whether their size exceeds the sample mean and conducts separate regression analyses. The regression results are presented in Table 11, where Column (1) presents the econometric regression results for the large enterprise sample, and Column (2) presents those for the small enterprise sample.

**Table 11:** Heterogeneity results by firm size.

Variables	Large enterprises	Small enterprises
	(1)	(2)
Covid19_t_*ASE_i_	6.901^***^ (2.349)	0.356 (1.393)
Control variables	Yes	Yes
Firm FE	Yes	Yes
Year FE	Yes	Yes
observations	14,416	16,644
Adj R-square	0.713	0.803

The values in parentheses represent robust standard errors clustered at the firm level. *, *, and *** denote significance at the 10, 5, and 1% levels, respectively.

Based on the results in Column (1), it is evident that in large enterprises, following the impact of the COVID-19 shock, private enterprises with administrative embedding significantly enhanced employee health insurance coverage. In contrast, from the results in Column (2), it is evident that in small enterprises, private enterprises with administrative embedding did not significantly improve employee health insurance coverage after the COVID-19 shock. The results, accounting for the heterogeneity of firm size, demonstrate that in large private enterprises, the impact of the COVID-19 shock and administrative embedding on enhancing employee health insurance coverage is more significant. On the one hand, this is because large enterprises possess greater capacity and willingness to improve employee health insurance coverage to attract and retain talent, thereby enhancing their corporate image. On the other hand, large enterprises may place greater emphasis on administrative embedding, and the administrative embedding within these enterprises is more effectively able to fulfill its role, leading to a more significant impact on enhancing employee health insurance coverage.

## 6 Mechanism test and further analysis

### 6.1 Mediating effect

Administrative embedding plays a crucial role in guiding and supervising enterprises to undertake more and better social responsibilities. Out of concern and emphasis on employee rights, enterprises may pay more attention to employee training, which helps companies better understand production safety knowledge and health protection arrangements. This, in turn, reduces the likelihood of safety disputes with employees and minimizes the possibility of layoffs. By enhancing attention to employee health protection, employees can work with peace of mind. Therefore, we tested the possible internal supervision mechanisms through which administrative embedding affects employee health protection. Specifically, we examined the impact of administrative embedding on employee training, employee safety disputes, and layoffs. Among them, whether the enterprise employees have received vocational training is a binary variable. If the employees have received vocational training, the value is 1, otherwise, the value is 0. Whether an employee has a safety dispute is a binary variable. If an employee in the enterprise has a safety dispute, the value is 1, otherwise, the value is 0. Enterprise layoffs are a binary variable. If an enterprise has carried out layoffs, the value is 1, otherwise, the value is 0. All the data used are sourced from the CSMAR database. The test results are shown in Table 12. The findings suggest that administrative embedding significantly improves employee training, reduces safety disputes, and decreases layoffs. This indicates that administrative embedding enhances corporate attention to employee safety and health, and internal supervision is an important mechanism through which administrative embedding promotes the improvement of employee health protection levels.

**Table 12:** Results of mediating effect.

Variables	Vocational training	Employee safety disputes	Layoffs
	(1)	(2)	(3)
Covid19_t_*ASE_i_	0.042^***^ (0.007)	−0.003^**^ (0.001)	−0.030^***^ (0.006)
Control variables	Yes	Yes	Yes
Firm FE	Yes	Yes	Yes
Year FE	Yes	Yes	Yes
observations	15,025	15,025	15,025
Adj R-square	0.432	0.551	0.465

The values in parentheses represent robust standard errors clustered at the firm level. *, *, and *** denote significance at the 10, 5, and 1% levels, respectively.

### 6.2 Moderating test

#### 6.2.1 Medical conditions

Considering that regional medical conditions may significantly influence the promotion of employee rights protection through administrative embedding, this study tests the moderating effect of regional medical conditions. The test results are presented in Table 13. Specifically, this study examines the moderating effect of urban medical conditions by constructing interaction terms between the impact of the COVID-19 shock, corporate administrative embedding, and the moderating variables. The data at the city level is matched according to the city where the enterprise is located. The number of hospitals, the number of hospital beds, and the number of assistant practicing physicians are all continuous variables. We take their natural logarithms, respectively, and add them to the regression equation for regression tests. The data used is sourced from the CSMAR database. The selected moderating variables include the number of hospitals, the number of hospital beds, and the number of licensed assistant physicians. The results indicate that regardless of whether the moderating variable is the number of hospitals, hospital beds, or licensed assistant physicians, the coefficients of the interaction terms between the COVID-19 impact, corporate administrative embedding, and the moderating variables are all significantly positive. This suggests that in cities with better medical conditions, the effect of corporate administrative embedding on improving employee health protection is stronger. Regional medical conditions positively moderate the enhancement of employee health protection through administrative embedding. In cities with better medical conditions, companies have a greater capacity to improve employee health protection, and the positive effects of administrative embedding are more readily realized, yielding more substantial results.

**Table 13:** Moderating effect of medical conditions.

Variables	Number of hospitals	Number of hospital beds	Number of licensed assistant physicians
	(1)	(2)	(3)
Covid19_t_*ASE_i_*moderator	0.032^***^ (0.012)	0.048^***^ (0.011)	0.056^***^ (0.011)
Covid19_t_*ASE_i_	−0.082 (0.062)	−0.422^***^ (0.117)	−0.487^***^ (0.111)
moderator	0.099^***^ (0.021)	0.256^***^ (0.037)	0.209^***^ (0.034)
Control variables	Yes	Yes	Yes
Firm FE	Yes	Yes	Yes
Year FE	Yes	Yes	Yes
observations	20,058	20,058	20,058
Adj R-square	0.759	0.760	0.760

The values in parentheses represent robust standard errors clustered at the firm level. *, *, and *** denote significance at the 10, 5, and 1% levels, respectively.

#### 6.2.2 Social security

The robustness of the urban social security system plays a moderating role in enhancing employee health protection through administrative embedding. A more comprehensive urban social security system reflects higher levels of urban governance and public services, enhanced protection of workers’ rights, and increased willingness and capacity of enterprises to improve employee health protection. Therefore, this paper investigates the moderating role of urban social security in the process by which administrative embedding enhances employee health protection. The test results, presented in Table 14, utilize urban basic medical insurance, basic pension insurance, and unemployment insurance as indicators to measure the level of urban social security. Urban basic medical insurance is measured by the natural logarithm of the number of participants in the basic medical insurance for urban employees, basic pension insurance is measured by the natural logarithm of the number of participants in the basic pension insurance for urban employees, and unemployment insurance is measured by the natural logarithm of the number of participants in unemployment insurance. The data used is sourced from the CSMAR database. The test results demonstrate that the coefficients of the interaction terms for public health shocks, enterprise administrative embedding, and the moderating variables are significantly positive. This suggests that in cities with higher social security levels, administrative embedding exerts a stronger influence on improving employee health protection, and urban social security plays a critical positive moderating role in this process.

**Table 14:** Moderating effect of social security.

Variables	Basic medical insurance	Basic pension insurance	Unemployment insurance
Covid19_t_*ASE_i_*moderator	0.040^***^ (0.006)	0.052^***^ (0.007)	0.039^***^ (0.006)
Covid19_t_*ASE_i_	−0.495^***^ (0.093)	−0.682^***^ (0.103)	−0.462^***^ (0.085)
moderator	0.028^***^ (0.008)	0.046^***^ (0.010)	0.056^***^ (0.013)
Control variables	Yes	Yes	Yes
Firm FE	Yes	Yes	Yes
Year FE	Yes	Yes	Yes
observations	28,715	28,821	28,883
Adj R-square	0.718	0.718	0.717

The values in parentheses represent robust standard errors clustered at the firm level. *, *, and *** denote significance at the 10, 5, and 1% levels, respectively.

### 6.3 Further analysis

Employee health protection is a key aspect of corporate governance and an important indicator of a company’s social responsibility. As noted, administrative embedding plays a crucial role in motivating private enterprises to embrace social responsibilities in the post-COVID-19 era. This study also examines its impact on other facets of corporate social responsibility, as detailed in Table 15. Among them, the total donation amount is represented by the natural logarithm of the total enterprise donation amount. Whether the enterprise supports charity, whether the enterprise has carried out volunteer activities, whether the enterprise has provided international aid, whether the enterprise has driven employment, and whether the enterprise has promoted local economic development are all binary variables. If relevant behaviors have occurred, the value is 1; otherwise, the value is 0. All the data used are sourced from the CSMAR database. The results show that administrative embedding positively influences companies, leading to increased donations, stronger support for charitable causes, expanded volunteer efforts, greater international aid, improved employment opportunities, and enhanced local economic development. These findings highlight that administrative embedding promotes corporate social responsibility across various dimensions.

**Table 15:** Further analysis results.

Variables	Donation total	Supporting charity	Volunteer activities	International aid	Job creation	Promoting local economy
Covid19_t_*ASE_i_	0.337^***^ (0.054)	0.154^***^ (0.015)	0.060^***^ (0.018)	0.086^***^ (0.011)	0.173^***^ (0.018)	0.122^***^ (0.018)
Firm FE	Yes	Yes	Yes	Yes	Yes	Yes
Year FE	Yes	Yes	Yes	Yes	Yes	Yes
Observations	13,958	15,025	15,025	15,025	15,025	15,025
Adj R-square	0.776	0.623	0.573	0.625	0.510	0.528

The values in parentheses represent robust standard errors clustered at the firm level. *, *, and *** denote significance at the 10, 5, and 1% levels, respectively.

## 7 Conclusion and policy implications

This study uses the DID method to analyze privately-owned listed companies on China’s Shanghai and Shenzhen A-shares markets from 2011 to 2021, investigating the impact of public health shocks and administrative embedding on employee health protection. The results show that private enterprises with administrative embedding significantly improved employee health protection following public health shocks. Parallel trend tests, endogeneity treatments, and robustness checks confirm the results’ validity. This effect is stronger in enterprises located in eastern regions, areas with higher population density, and larger-scale firms. Administrative embeddings focus on employee welfare, coupled with urban medical and social security conditions, served as key mechanisms. After public health shocks, these organizations encouraged enterprises to take on greater social responsibility, leading to stronger attention to employee health, especially in companies that had previously neglected this area. Administrative embedding played a crucial role in uniting internal forces, fostering cohesion, and guiding enterprises in fulfilling their social responsibilities.

As the largest developing country globally, the findings of this study hold significant implications not only for China’s future policy formulation but also for corporate governance and high-quality development in other developing nations. Based on the research conclusions, the following policy recommendations are proposed:

Firstly, private enterprises should recognize the essential role of the mechanism of administrative embedding in enhancing corporate governance, promoting employee health protection, and fulfilling social responsibilities. To ensure its effective implementation, defining specific pathways and governance frameworks is crucial. The government should guide enterprises—especially Small and Medium Enterprises (SMEs)—to proactively participate in the mechanism of administrative embedding, ensuring their active involvement in corporate governance. Relevant supportive policies (e.g., tax incentives, financial subsidies) are recommended to incentivize enterprises, particularly in regions and industries with inadequate administrative embedding. Concurrently, enterprises need to optimize internal governance: the mechanism of administrative embedding should play a pivotal role in decision-making, strengthening oversight of employee health protection, compensation, and welfare through specialized committees or union assemblies.

Secondly, in the implementation of employee health protection policies, employee participation and empowerment are vital. The government should encourage enterprises to provide employees with decision-making authority in health protection matters, ensuring that they have sufficient opportunities to engage in the formulation and execution of policies. For instance, under the guidance of the mechanism of administrative embedding, enterprises can establish health committees or conduct regular health assessments to gather employees’ feedback and needs, allowing employees to actively contribute to workplace health discussions. The mechanism of administrative embedding can play a coordinating and supervisory role in this process, ensuring that employees’ voices are fully incorporated into health protection policies, thereby facilitating improvements in corporate health protection. In this way, employee health protection becomes not only a benefit derived from the policy but also a key driver of its effective implementation.

Thirdly, in the process of promoting the mechanism of administrative embedding, it is crucial to ensure both the efficiency and appropriateness of policy implementation. Excessive intervention can undermine the autonomy, innovation, and flexibility of enterprises, ultimately affecting their market competitiveness. Therefore, the government must precisely define the role of the mechanism of administrative embedding within enterprises to avoid excessive administrative interference, ensuring that enterprises’ decision-making power is not unduly constrained. At the same time, the mechanism of administrative embedding should prioritize execution efficiency to avoid resource wastage. The goal is to ensure that the mechanism of administrative embedding enhances employee health protection while avoiding excessive intervention, maintaining the flexibility and vitality of enterprises, and ultimately improving the overall efficiency and effectiveness of policy execution.

Fourthly, in the face of future public health crises, the mechanism of administrative embedding can significantly enhance the resilience of both enterprises and the state. By strengthening the connection between enterprises and the government, the mechanism of administrative embedding can provide timely policy support and facilitate resource mobilization, helping enterprises respond rapidly to crises, ensure the health and safety of employees, and maintain production. Simultaneously, the government can utilize the mechanism of administrative embedding to advance the development of public health systems, particularly by fostering a sense of social responsibility and enhancing public health involvement among enterprises during crises, thereby improving overall response speed. Furthermore, the government should strengthen and refine regional public health emergency mechanisms, ensuring the rapid allocation of resources and further improving the efficiency of emergency responses during public health crises.

## Data Availability

The data analyzed in this study is subject to the following licenses/restrictions: the data that support the findings of this study are available from CSMAR (https://data.csmar.com/). Restrictions apply to the availability of these data, which were used under license for this study. Data is available from the authors with the permission of CSMAR. Requests to access these datasets should be directed to https://data.csmar.com/.
